# Beta-Glucanemia after Coronary Artery Bypass Graft Surgery: A Case Report

**DOI:** 10.3390/jof4040114

**Published:** 2018-10-02

**Authors:** Ashley Styczynski, Hector Bonilla, Elizabeth Treynor, Jolly Shashank, Yonglong Zhang, Malcolm Finkelman

**Affiliations:** 1 Department of Infectious Disease, Stanford University Hospital, Palo Alto, California, CA 94305, USA; hbonilla@stanford.edu; 2 Washington Hospital, Fremont, California, CA 94538, USA; etreynor@gmail.com (E.T.); shashankjollyrmc@gmail.com (J.S.); 3 Research Laboratory, Clinical Development Department, Associates of Cape Cod, Falmouth, Massachusetts, MA 02536, USA; yzhang@acciusa.com (Y.Z.); MFinkelman@acciusa.com (M.F.)

**Keywords:** beta-glucan, CABG, blood conservation

## Abstract

Blood salvage techniques are increasingly being used during surgical procedures to reduce the need for exogenous blood products. The blood recovered from the surgical field through aspiration or absorption by surgical sponges is reinfused into a patient. A 65-year old patient who underwent coronary artery bypass grafting using blood salvage techniques developed a fever on post-op day 3 and was noted to have an elevated β-d-glucan level, a marker of systemic fungal infections. Ultimately, no fungal infection was identified, β-d-glucan levels slowly decreased and the patient demonstrated clinical improvement. To determine whether blood salvage procedures led to his elevated β-d-glucan levels, the surgical sponges were tested for elutable levels of β-d-glucan. The β-d-glucan content of the eluents was measured using the Fungitell® IVD kit (Associates of Cape Cod, Inc.; East Falmouth, MA). The β-d-glucan levels were found to be in concentrations 10,000-times greater than the limit of detection for human serum. While various studies have demonstrated both the immunomodulatory and pro-inflammatory effects of β-d-glucan, the physiologic impact of such high levels of β-d-glucan post-operatively remains unknown. Additionally, the persistence of detectable β-d-glucan up to several weeks after surgical procedures presents a challenge for the diagnosis of invasive fungal infections. Further studies are needed to assess the beta-glucanemia-related safety of surgical materials and their potential biological effects.

## 1. Introduction

Coronary artery bypass grafting (CABG) is a relatively common surgical procedure that has increased the demand for safe and cost-effective blood products. As a result, blood conservation techniques have become a mainstay of surgery where blood that has been aspirated from the surgical field or absorbed by surgical sponges can be re-infused into patients [1]. Although these methods are associated with the activation of coagulation and inflammatory systems, those effects have not been associated with increased morbidity and mortality [2,3].

A 65-year-old male with a history of autoimmune hemolytic anemia requiring corticosteroid therapy, myelodysplastic syndrome and diabetes mellitus, presented with unstable angina. On cardiac catheterization, he had severe coronary artery disease and underwent six-vessel CABG. During the surgery, blood conservation procedures were employed to minimize blood loss. The surgery proceeded uneventfully.

On post-op day 3, the patient developed a fever and leukocytosis. His exam was notable for Cushinoid features. He was normotensive without respiratory compromise. He had no signs of thrush. His surgical site was intact, and his cardiopulmonary exam was unremarkable. Intravenous sites were neither erythematous nor painful. The remainder of the physical exam was benign.

The patient’s concurrent laboratory findings demonstrated leukocytosis and mild thrombocytopenia. Infectious disease testing revealed serum β-d-glucan levels above the limit of detection (>500 pg/mL), but galactomannan was undetectable. A computed tomography (CT) scan of the chest revealed an apical spiculated nodule that was stable compared to prior scans.

The patient was empirically treated with ceftriaxone but had received no other new medications or infusions, and his fever and leukocytosis improved. Blood and urine cultures remained negative. We suspected that the elevated β-d-glucan was related to the use of surgical sponges and gauze as part of blood conservation. Therefore, he did not receive any anti-fungal therapy. The patient had a satisfactory recovery and was discharged on hospital day 7. Subsequent β-d-glucan levels showed delayed normalization with levels of 271 pg/mL on post-operative day 13 and was undetectable on post-operative day 52.

Fungitell® is an FDA-approved serum biomarker for the clinical diagnosis of fungal infections, mainly *Candida* and *Aspergillus* species. This assay is based on detection in serum of (1→3)-β-d-glucan with a modified *Limulus* amoebocyte lysate test [4]. The sensitivity and specificity of the test for detecting invasive fungal infections (IFI) is estimated at 73% and 81%, respectively, based on a cutoff value of 80 pg/mL [5]. This is considered superior detection to the current gold standard of blood cultures that have a lower sensitivity of approximately 50% [6]. However, clinical false-positives for β-d-glucan have been observed in patients undergoing hemodialysis with cellulose membranes [7], being treated with immunoglobulin products [8,9], and, rarely, being exposed to gauze or related materials [1], among other etiologies [10,11,12].

## 2. Materials and Methods

To confirm our suspicion regarding the source of the patient’s elevated β-d-glucan levels, samples of the surgical sponges and gauzes were evaluated for elutable β-d-glucan by the Research Laboratory, Clinical Development Department, Associates of Cape Cod, Inc. The sponges and gauzes were soaked in β-glucan-free water for approximately 22 hours. The β-d-glucan content of the extracts was measured using the Fungitell in vitro diagnostic kit (Associates of Cape Cod, Inc.; East Falmouth, MA) according to the manufacturer’s instructions.

## 3. Results

The quantities of β-d-glucan leached from the sponges and gauzes were found to be more than 10,000 times greater than the upper limit of detection for serum-based assays with a mean of 4.853 μg/mL and 6.596 μg/mL, respectively (Table 1). This is in contrast with the clinical interpretation of negative (<60 pg/mL), indeterminate (60–79 pg/mL), and positive (≥80 pg/mL) threshold results, though the interpretation is limited by unknown fluid extraction volumes and mass of the sponges and gauze.

## 4. Discussions

We report a patient who underwent CABG surgery with a post-operative course that was complicated by fever and leukocytosis, as well as elevated β-d-glucan levels with risk factors for IFI, including chronic steroid use and diabetes. We confirmed our suspicion that the elevated β-d-glucan levels reflected blood salvage techniques during surgery, rather than IFI. While the diagnostic limitations of β-d-glucan for detecting IFI have been previously reported, such as occurred in our patient, what is less clear is the biological significance of elevated circulating β-d-glucan levels. β-d-glucan-containing medical products have been shown to have anti-infectious immunity and anti-carcinogenic activity [7,13,14,15]. β-d-glucan exposure has also demonstrated immunomodulatory effects in healthy individuals, such as attenuated CD86 and CD80 expression and a reduction in inflammatory cytokines IL-2, IL-8, IL-12, TNF-α, and IFN-γ [16].

In contrast, systemic β-d-glucan exposure has demonstrated the activation of inflammatory processes in animal models and cell cultures [15,16,17,18]. Co-exposure to β-d-glucan and toll-like receptor agonists in human whole blood models increased pro-inflammatory cytokines IL-6 and IL-8 [19]. Additionally, prior systemic exposure to β-d-glucan followed by sub-lethal bacterial endotoxin exposure elicited fatal shock-like responses in rodent models [20,21]. We did not measure circulating cytokines for our patient, so were unable to assess whether his biological markers correlated with his clinical findings.

Elevated β-d-glucan may be common after surgical procedures where these types of surgical materials and blood conservation protocols have been used. The presence and persistence of detectable β-d-glucan up to several weeks after some surgical procedures presents a challenge for the diagnosis of IFI, particularly in patients such as this one who may have other risk factors for IFI. Further, the short-term or long-term implication of this load of β-d-glucan in the circulation remains unknown. Prospective and comparative studies are needed to assess beta-glucanemia-related safety of surgical materials and their potential biological effects.

## Figures and Tables

**Table 1:** In vitro quantification of (1→3)-β-d-glucan amount from surgical sponges and gauzes.

Safe-T Lap *	Sponge 1	Sponge 2	Sponge 3	Mean
β-glucan (μg/mL)	5.387	4.665	4.507	4.853
X-ray Detectable *	Gauze 1	Gauze 2	Gauze 3	Mean
β-glucan (μg/mL)	3.437	4.394	11.957	6.596

* SurgiCount Medical, Irvine, CA. The results of β-d-glucan of the eluents from the surgical sponges and gauzes are presented in triplicate along with the mean values for each.
